# Editorial: Women in science—precision medicine 2023

**DOI:** 10.3389/fmed.2026.1927814

**Published:** 2026-08-19

**Authors:** Geraldine H. O'Sullivan Coyne, Alice P. Chen

**Affiliations:** 1 START Center for Cancer Research, New York, NY, United States; 2 AP Chen Consultant, Potomac, MD, United States

**Keywords:** bioinformatics, female, genomics, overactive bladder, precision medicine

For 2025, the NIH's National Human Genome Research institute defined precision medicine as “an innovative approach that uses information about an individual's genomic, environmental and lifestyle information to guide decisions related to their medical management.” We are agreed that this type of definition immediately makes us think of the sheer, and daunting, amount of information required to generate “precision” for patients; and the effort involved in creating customization of healthcare. It is an intuitive concept, as no two human beings are alike, and could be argued dates back to ancient Greece with Hippocrates' exhortation to “treat the patient not the disease.” Tailoring care uniquely to an individual has increasingly involved analysis of large datasets (whether genomic or clinical) with intricate stakeholders ranging from industry to academia and technology, and with consequent transformative regulatory and ethical corollaries. This focus on producing accurate results that give both patients and physician confidence to pursue a customized clinical decision has impulse forward the field of multiple scientific specialties.

This Research Topic focuses on highlighting leading women from all corners of the world that are engaged in exploring the continuously evolving frontier of precision medicine. Women have a long history of leading innovation and research in medicine, and we are delighted to bring this selection of six articles to celebrate the continued contributions of female leaders to science. The majority of these articles are in the field of oncology, not unexpected given the continued relevance of identifying new targets for oncology drug development which relies heavily on precision medicine. The impact of precision medicine across the breath of oncology is also nicely highlighted given these articles address issues in the more common malignancy that is hormone positive breast cancer 1 vs. the much rarer glioblastoma multiforme 2. In these articles (Luo et al., Linehan et al., Pollard et al., Ghanem et al.), the use of precision genomic bioinformatics testing, both somatic and germline, is employed to target specific molecular characteristics of a disease in improve outcomes for patients. It is also hard not to recognize the impact that these types of analysis have on the development of new clinical trials, both of novel targeted agents alone or in combination with others. We hope that you will recognize how precision strategies in each of these articles, whether from biomarker testing, clinical databases or clinical trials, result in a wealth of information for analysis and validation, and for kickstarting new hypotheses. Our leading female authors in each of these diverse articles within the field of medical oncology strive to improve patient outcomes in life threatening malignancies.

However, precision medicine as mentioned above, does also extend to other fields: such a utilizing a personalized surgical intervention in a topic very relevant to female healthcare and marking a thoughtful addition to pursing change in this field toward more equitable care through updated treatment paradigms as noted of Tan et al.'s article on overactive bladder management with electroacupuncture and Gao's management of temporomandibular diseases.

Reflecting on 2021's topic of Women in Science-Precision Medicine, it is clear that women continue to lead and impact in this area. A number of groups across the world, whether academic or industry based, have developed specific endeavors, collectives, or platforms to highlight and advance not only women's achievements in science and technology but also science and outcomes that empower women's health outcomes. March 8th marks International Women's Day—a day to remember the courage and leadership of women throughout the years, and their contribution to inspiring the next generation of leaders and scientists.

